# Enhanced 3D visualization for planning biventricular repair of double outlet right ventricle: a pilot study on the advantages of virtual reality

**DOI:** 10.1093/ehjdh/ztab087

**Published:** 2021-10-28

**Authors:** Elena Giulia Milano, Martin Kostolny, Endrit Pajaziti, Jan Marek, William Regan, Massimo Caputo, Giovanni Battista Luciani, Kristian H Mortensen, Andrew C Cook, Silvia Schievano, Claudio Capelli

**Affiliations:** 1 UCL Institute for Cardiovascular Science and Great Ormond Street Hospital, 20c Guilford St, London WC1N 1DZ, UK; 2 Department of Surgery, Dentistry, Paediatrics and Gynaecology, University of Verona, P.le Scuro 10, 37134, Verona, Italy; 3 Department of Cardiothoracic Surgery, Great Ormond Street Hospital for Children NHS Foundation Trust, Great Ormond Street, WC1N 3JH, London, UK; 4 Cardiorespiratory Division, Great Ormond Street Hospital for Children NHS Foundation Trust, Great Ormond Street, WC1N 3JH, London, UK; 5 Department of Congenital Heart Disease, Evelina London Children’s Hospital, Westminster Bridge Rd, SE1 7EH, London, UK; 6 Bristol Heart Institute, Bristol Medical School, Bristol Medical School, University of Bristol, St Michael's Hill, BS2 8DZ, Bristol, UK

**Keywords:** Congenital heart disease, Double outlet right ventricle, Cardiac computed tomography, 3D printing, Virtual reality

## Abstract

**Aims:**

We aim to determine any additional benefit of virtual reality (VR) experience if compared to conventional cross-sectional imaging and standard three-dimensional (3D) modelling when deciding on surgical strategy in patients with complex double outlet right ventricle (DORV).

**Methods and results:**

We retrospectively selected 10 consecutive patients with DORV and complex interventricular communications, who underwent biventricular repair. An arterial switch operation (ASO) was part of the repair in three of those. Computed tomography (CT) or cardiac magnetic resonance imaging images were used to reconstruct patient-specific 3D anatomies, which were then presented using different visualization modalities: 3D pdf, 3D printed models, and VR models. Two experienced paediatric cardiac surgeons, blinded to repair performed, reviewed each case evaluating the suitability of repair following assessment of each visualization modalities. In addition, they had to identify those who had ASO as part of the procedure. Answers of the two surgeons were compared to the actual operations performed. There was no mortality during the follow-up (mean = 2.5 years). Two patients required reoperations. After review of CT/cardiac magnetic resonance images, the evaluators identified the surgical strategy in accordance with the actual surgical plan in 75% of the cases. When using 3D pdf this reached only 70%. Accordance improved to 85% after revision of 3D printed models and to 95% after VR. Use of 3D printed models and VR facilitated the identification of patients who required ASO.

**Conclusion:**

Virtual reality can enhance understanding of suitability for biventricular repair in patients with complex DORV if compared to cross-sectional images and other 3D modelling techniques.

## Graphical Abstract

**Figure:**
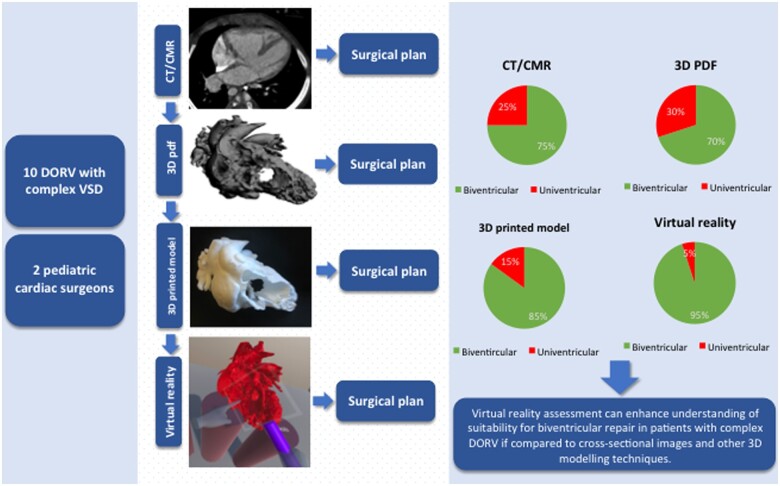

## Introduction

Double outlet right ventricle (DORV) represents a wide range of anatomical configurations that, together with associated abnormalities, often result in unique anatomies requiring individualized surgical repair. The presence of non-committed or remote interventricular communications [ventricular septal defect (VSD)] represents a particular surgical challenge and often drives the choice towards a univentricular repair, even in the setting of adequately sized left ventricle (LV) and right ventricle (RV).^1^

The initial DORV sub-type influences patient outcomes: non-committed VSD is associated with early and late mortality^2^ while an unfavourable intracardiac tunnel geometry may lead to left ventricular outflow tract obstruction (LVOTO).^3^ Intracardiac tunnelling with arterial switch operation (ASO) carries the highest risk of early mortality.^2^

Advanced visualization techniques are currently available to appreciate the images of patients fully in three dimension (3D). Techniques now include visual patient-specific 3D modelling; 3D printing, augmented and virtual reality (VR) derived from conventional diagnostic image modalities such as cardiac magnetic resonance (CMR) and computed tomography (CT).

Over the last decade, the use of 3D printing in complex congenital heart disease, and particularly in DORV, has gained importance in surgical and interventional procedural planning,^4–8^ and anatomical evaluation.^9–11^ However, access to this technology is still limited by the associated costs, timing, printer accessibility, thus leading to incompatibility with the workflow of clinical care.

Three-dimensional model on screen, such as the 3D pdf file format, allows clinicians to visualize and to handle the 3D patient anatomy on any screen, computer, or mobile device, without the need for technical expertise in software navigation.

Virtual reality is an immersive technique offering the possibility of navigating and manipulating patient-specific anatomies, thus potentially overcoming some of the limitations of 3D printing. Recent results of the use of VR for visualization and analysis of congenital heart disease^12–14^ are promising with or without preliminary image segmentation.^15^ However, it has not been assessed yet whether there are any potential improvement in using VR to plan the surgical workup of patients with DORV.

We present a pilot study focused on a series of DORV patients operated at our Centre to assess the role of different 3D modelling techniques (3D pdf, printed models, and VR) and conventional cross-sectional imaging aiming to determine which modality is most helpful in tailoring the surgical strategy.

## Methods

### Patient population and image data

We retrospectively selected 10 consecutive patients with complex DORV who successfully underwent biventricular repair with intracardiac baffle, with or without arterial switch, between August 2015 and March 2018 at our Institution. The surgical plan was formulated after a joint discussion among cardiologist and cardiac surgeons and reviews of cross-sectional echocardiography and cardiac CT or magnetic resonance imaging (MRI) data. All procedures were carried out by a senior surgeon who accessed a 3D patient-specific reconstruction before surgery. The surgeon had the possibility to manipulate the model to search for the most informative view. The relevant clinical information, including post-operative follow-up, and cross-sectional imaging data (CT or CMR) were collected for all patients.

Computed tomography images were acquired using dual-source multidetector CT scan (Siemens Somatom Force, Siemens Healthineers, Erlangen, Germany), as contrast-enhanced non-ECG-gated datasets.

Cardiac MRI was acquired with contrast-enhanced 3D balanced steady-state free precession (3D whole heart) in mid-diastole with respiratory navigator at 1.5 Tesla (Siemens Avanto).

The study was approved by the local R&D office.

### Patient-specific three-dimensional anatomical model reconstructions

Volumetric images were post-processed by means of a commercially available software (ScanIP, Synopsis-N2018.03) to reconstruct the 3D anatomy of the heart including atria, ventricles, great vessels, and, where possible, valvular structures. The segmentation process was performed by the same operator (C.C.) with over 10 years of experience. The reconstruction of each model took an average of 2.5 h. The resulting 3D anatomical reconstructions were imported as *.stl* file format into Meshmixer (2017 Autodesk, v3.5). For 3D printing, two planar cuts were performed, one across the RV free wall and one across the LV posterior wall, to expose the intracardiac anatomy and the VSD. The cuts were indicated by the operating surgeons as optimal to evaluate the patient-specific anatomies. A 1 mm homogenous thickness was added to each cardiac surface to allow for manufacturing.

### Three-dimensional visualization tools

Following the post-processing, the 3D pdf file of each patient model was exported a built-in using ScanIP. The 3D pdf file included the same planar cuts of the 3D printed model.

All models were printed at 1:1 scale, in rigid white nylon (EOS PA2200 Nylon 12) using selective laser technology (EOS P100).

The 3D reconstructions (as .obj files) were imported into a novel VR environment developed in-house within the Unity engine. The target platform was the Oculus Rift system (comprised of a headset, two sensors, and two hand controllers). The following specialized VR tools were specifically designed for this study: (i) grabbing tool to hold and rotate the object; (ii) cutting tool for slicing and exposing the intracardiac anatomy in any plane; (iii) measuring tool to measure distance and diameters of the different cardiac structures; (iv) marking tool to place landmark on the virtual model; (v) multiplanar reconstruction tool to crosscut the model in three different perpendicular planes (axial, sagittal, coronal); and (vi) virtual echo tool to scan the model with a virtual probe in any desirable plane. The user could freely interact, move, and rotate each patient-specific model in virtual space and use any of the aforementioned tools.

### Evaluation of patient model and surgical strategy

**Figure 1:**
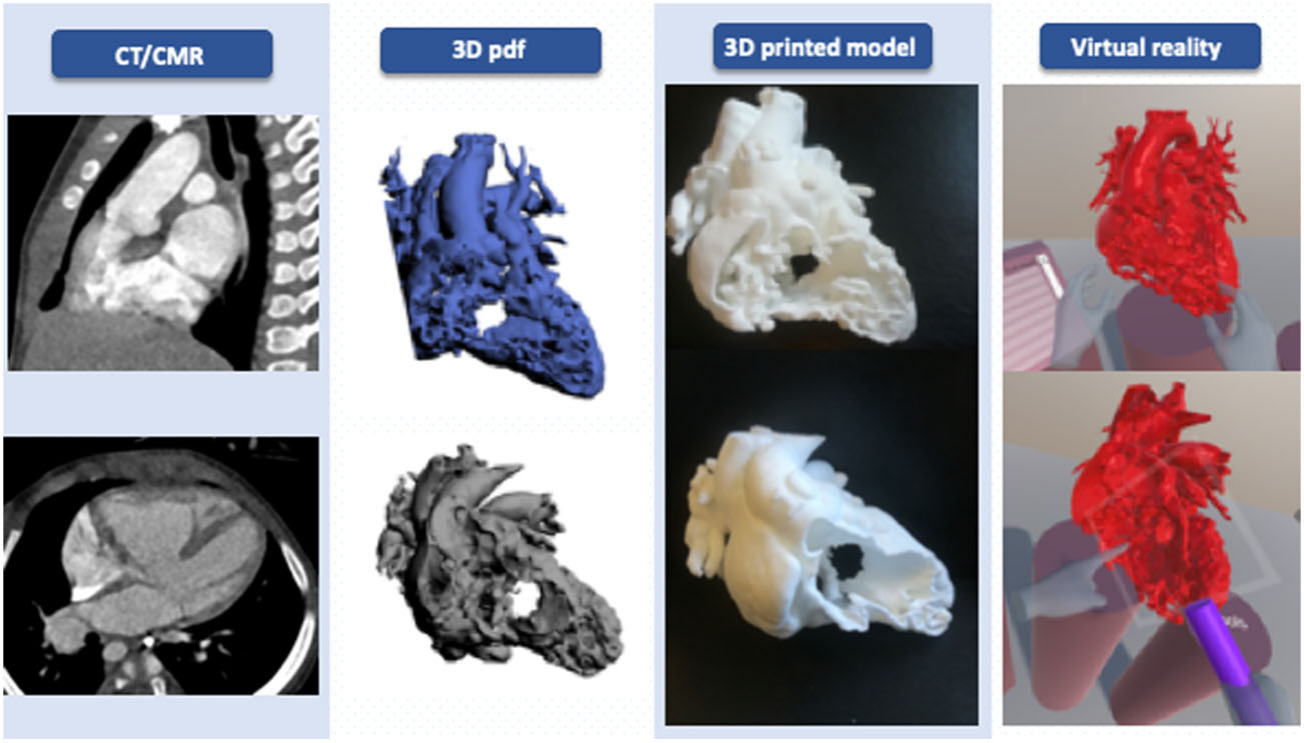
Two examples of three-dimensional visualization tools used by Surgeon A and B to assess the cases. CMR, cardiac magnetic resonance; CT, computed tomography; 3D, three-dimensional.

Each case was retrospectively evaluated, separately, by two experienced paediatric cardiac surgeons, from different centres, each with >15 years of experience as first operator in paediatric cardiac surgery. They were neither involved in the original surgery nor with the clinical care of the patients and were completely blinded to the actual surgical repair and outcome. The two surgeons were asked to provide a surgical plan for each case retrospectively analysed, using four different visualization tools. In particular, their decision on the type of repair (biventricular vs. univentricular) was recorded at the end of each stage of the analysis. The first assessment of patients was based on the clinical history, and conventional imaging modalities such as echocardiography and CT/CMR images. This review of the CT/CMR images was guided by a cardiologist with experience in cardiovascular imaging. Hence, the surgeons were presented sequentially with patient-specific 3D models of each case in form of (i) a 3D pdf; (ii) a physical 3D printed model; and (iii) the VR setup (*Figure  1*).

At each step, the surgeons were asked to confirm or change their potential surgical approach. Each decision was compared to the actual strategy performed on each patient (i.e. the choice of reference) in order to evaluate the accuracy of each type of 3D modelling modality in planning complex surgical repairs. The actual surgical strategy was considered successful for each patients based on their positive outcome within their follow-up time.

## Results

### Patient population

Patient baseline characteristics are summarized in *Table 1*. Nine patients had previous palliation at a median of 0.6 months (0.1–3) including pulmonary artery banding (PAB, *n* = 6), arch repair and PAB (*n* = 2), and bilateral PAB and patent duct arteriosus stent (*n* = 1). In accordance with the plan agreed by our multidisciplinary team, all patients underwent biventricular repair with intracardiac tunnelling ± VSD enlargement at a median of 8.4 months (2.6–13.8). In one case, an intraoperative decision to perform an ASO as part of the repair had to be made. In three cases (Case 1, Case 6, Case 7), ASO was performed. Details of each procedure and left ventricular outflow tract (LVOT) gradients at discharge are recorded in *Table 2*.

**Table 1:** Patient baseline characteristics

Patient case	Imaging modality	Location of interventricular communication	Arrangement of great arteries	Additional findings
1	CT	Multiple (non-committed + apical)	Parallel, aorta anterior and to right	Atrial septal defect, aortic arch hypoplasia, aortic coarctation
2	CMR	Subpulmonary	Parallel, side by side, aorta to right	Patent foramen ovale, aortic coarctation, RCA from left facing sinus
3	CT	Non-committed	Parallel, side by side, aorta to right	—
4	CT	Non-committed	Parallel, side by side, aorta to right	Large VSD split in two by large muscular bridge
5	CT	Double committed with inlet extension	Parallel, side by side, aorta to right	Sub-aortic narrowing, anomalous left anterior descending from RCA
6	CT	Non-committed	Parallel, side by side, aorta to right	Large conal branch running on the anterior wall of RV
7	CT	Multiple (non-committed + small muscular)	Parallel, aorta anterior and to right	—
8	CT	Non-committed	Parallel, side by side, aorta to right	Subpulmonary and main pulmonary artery stenosis
9	CT	Non-committed	Parallel, aorta anterior and to right	Atrial septal defect, chordal attachment of tricuspid valve to interventricular septum
10	CT	Non-committed	Parallel, aorta anterior and to right	Aortic arch hypoplasia, aortic coarctation

CMR, cardiac magnetic resonance; CT, computed tomography; RCA, right coronary artery; RV, right ventricle; VSD, ventricular septal defect.

**Table 2:** Procedural data

Patient case	Type of Bi-V repair	Previous palliation	Age at palliation (months)	Age at repair (months)	Weight at repair (kg)	Discharge LVOT (m/s)
1	ASO, intraventricular tunnel, and arch repair	Bilateral PAB and PDA stent	0.7	6	5.5	1.2
2	Intraventricular tunnel	Arch repair, PAB	0.1	11	9.4	1.1
3	Intraventricular tunnel	PAB	1.4	7	6.4	1.4
4	Intraventricular tunnel	PAB	0.5	5	6.4	2.2
5	Intraventricular tunnel	PAB	2.0	3	4.7	1.2
6	ASO and intraventricular tunnel	PAB	2.6	10	8.6	1.4
7	ASO and intraventricular tunnel	PAB	0.4	13	8.0	2.5
8	Intraventricular tunnel	NA	NA	5	7.5	1.3
9	Intraventricular tunnel	PAB	3.0	11	7.9	1.2
10	Intraventricular tunnel	Arch repair, PAB	0.1	10	9.1	1.1

ASO, arterial switch operation; Bi-V, biventricular; CPB, cardiopulmonary bypass; LVOT, left ventricular outflow tract; NA, not applicable; PAB, pulmonary artery banding; PDA, patent duct arteriosus.

The median follow-up was 31 months (10.2–44.6). There was no mortality during follow-up. Two patients underwent further surgical procedures. Patient #1 had surgical closure of residual apical VSDs due to the persistence of significant left to right shunt 24 months after initial repair with arterial switch, arch repair, and intracardiac tunnel with limitation of pulmonary blood flow by creating a supravalvar pulmonary stenosis (Case 1). Patient #7 underwent two reoperations for recurrent LVOTO. A resection of a fibro-muscular shelf was performed at 8 months after repair and later replacement of VSD patch at second reoperation at 23 months. One more patient (i.e. Patient #5) is currently scheduled for reoperation with an LVOTO gradient of 4.1 m/s 40 months after the biventricular repair. Follow-up data such as LVOT gradients are summarized in *Table 3*.

**Table 3:** Follow-up data

Patient case	Time of FU (months)	Reoperations during FU	LVOT V max (m/s)	RVOT V max (m/s)
1	44.6	Closure of multiple apical VSDs	1.5	1.7
2	33.6	No	1.5	1.5
3	40.5	No	3.3	2.1
4	28.6	No	2.2	2.4
5	39.9	No	4.1	1.9
6	43.8	No	1.5	1.5
7	23.0	LVOTO relief (2×)	1.5	1.3
8	10.2	No	1.3	2
9	24.2	No	1.3	1.7
10	16.0	NO	1.3	1.5

FU, follow-up; LVOT, left ventricular outflow tract; LVOTO, left ventricular outflow tract obstruction; RVOT, right ventricular outflow tract; VSD, ventricular septal defect.

### Surgical planning

The choices of the two surgeons interviewed were evaluated if in agreement or not with the operation performed (*Table 4*). After review of the CT or CMR data, a biventricular repair strategy was in agreement with the actual procedure in 9/10 cases by *Surgeon A* and in 6 out of 10 cases by *Surgeon B*.

**Table 4:** Agreement with chosen surgical strategy (arterial switch operation: *Yes* vs. *No*) according to different three-dimensional tools: the tick (✓) shows the cases when the surgeon correctly identified the need for arterial switch operation; the cross (×) shows the cases when the surgeon did not correctly identified the need for arterial switch operation

	ASO		CT/CMR	3D PDF	3D print	VR
1	Yes	surgA	**✓**	**✓**	Uni-V repair	**✓**
		surgB	Uni-V repair	Uni-V repair	Uni-V repair	×
2	No	surgA	**×**	**×**	**×**	**×**
		surgB	**×**	**×**	**×**	**×**
3	No	surgA	**×**	**×**	**✓**	**✓**
		surgB	**✓**	**✓**	**×**	**✓**
4	No	surgA	**✓**	**×**	**×**	**×**
		surgB	**×**	**×**	**✓**	**×**
5	No	surgA	**×**	**✓**	**✓**	**✓**
		surgB	**×**	**×**	**×**	**×**
6	Yes	surgA	**✓**	**✓**	**✓**	**✓**
		surgB	**✓**	**✓**	Uni-V repair	Uni-V repair
7	Yes	surgA	**✓**	**✓**	**✓**	**✓**
		surgB	Not sure	Not sure	**✓**	**✓**
8	No	surgA	**✓**	**✓**	**✓**	**✓**
		surgB	**✓**	**✓**	**✓**	**✓**
9	No	surgA	Uni-V repair	Uni-V repair	**✓**	**✓**
		surgB	Uni-V repair	Uni-V repair	Not sure	**✓**
10	No	surgA	**✓**	**✓**	**✓**	**×**
		surgB	Not sure	Not sure	**✓**	**✓**
TOT		—	9/20 (45%)	9/20 (45%)	11/20 (55%)	12/20 (60%)

ASO, arterial switch operation; CMR, cardiac magnetic resonance; CT, computed tomography; Uni-V, univentricular; VR, virtual reality; 3D, three-dimensional.

Following review of the 3D pdf models, *Surgeon A* did not change surgical strategy for any case (9/10 biventricular strategy), while *Surgeon B* modified the surgical plan for Patient 6 (5/10 biventricular strategy).

Following the review of the 3D printed model, biventricular repair was suggested in 9 and 8 cases out of 10 by *Surgeon A* and *Surgeon B*, respectively.

Using the VR setup, the concordance of choices increased to 10 out of 10 (*Surgeon A*) and 9 out of 10 (*Surgeon B*).

The agreement between the two observers increased from 70% after cross-sectional imaging review, to 90% following 3D printed model and VR review.

When a univentricular repair strategy was suggested this was due to the distance between the aortic and pulmonary annulus and the VSD.

**Figure 2:**
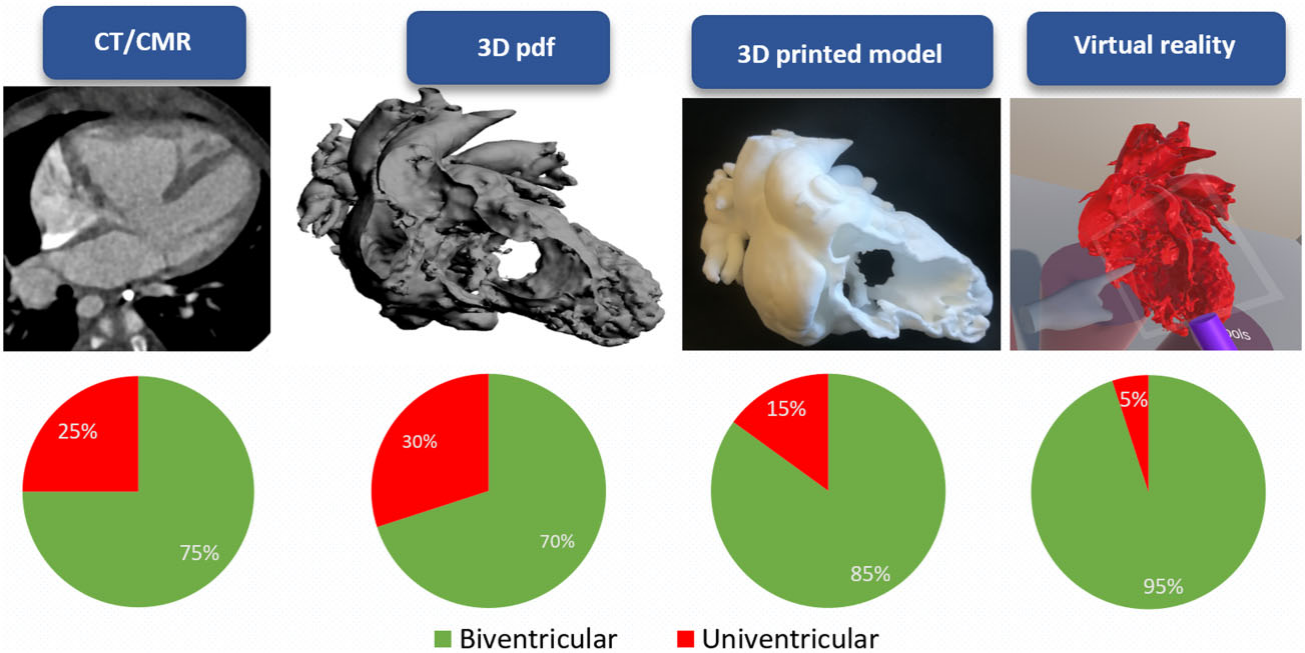
Visual summary of the level of accordance (%) between the two surgeon evaluators and the actual performed plan in relation to the imaging modality used. CMR, cardiac magnetic resonance; CT, computed tomography; 3D, three-dimensional.

Compared to traditional cross-sectional imaging, the 3D pdf did not increase the accordance on the feasibility of biventricular repair in any case. The agreement with the actual surgical plan improved in four cases with the use of the physical 3D printed model and in five cases using the VR setup, changing the surgical plan from univentricular to biventricular repair. The percentage of correct answers after reviewing all imaging modalities is summarized in *Figure  2*.

The identification of ASO as part of the repair strategy was in accordance with the original surgical plan in 6/10 cases for *Surgeon A* and in 3/10 cases by *Surgeon B* after review of CT/CMR images. For both surgeons, there was no improvement after reviewing the 3D pdf.

The accuracy increased after the evaluation of the physical 3D printed model: *Surgeon A* and *Surgeon B* correctly suggested if an ASO was needed or not in 7/10 and 4/10 patients, respectively. This did not change after VR assessment for *Surgeon A* but improved for *Surgeon B* from 4/10 to 5/10.

## Discussion

Three-dimensional modalities to visualize cardiac anatomy and plan surgical approaches are increasingly used in clinical practice. Over the last decade, 3D models including computer and 3D printed replica have shown both benefits and limitations. More recently, novel and more immersive ways to explore and interact with cardiac anatomy such as VR have been proposed. With the increasing use of these technologies, the aim of this study was to compare such 3D modalities in the context of repair of a complex of DORV. To the best of our knowledge, this is the first pilot study aiming to compare the influence of all the 3D modelling techniques available in the presurgical planning of complex congenital heart disease. The main findings of our study are the following:

1. The use of a 3D model on screen (i.e. 3D pdf) did not provide additional support in the selection and planning for patients suitable for biventricular repair procedure, compared to traditional cross-sectional imaging.
2. Three-dimensional printed models increased the accuracy in identifying the patients suitable for biventricular repair and in deciding about whether to perform ASO in the context of biventricular repair, compared to traditional cross-sectional imaging and also to 3D pdf.
3. Virtual reality assessment represents the best 3D modelling modality in the planning of DORV repair, increasing the accuracy of the selection of ideal candidates for biventricular repair to 95% (19/20 cases), compared to 75% (15/20 cases) of the traditional cross-sectional imaging techniques.

The role of 3D printing technology has become increasingly recognized in the evaluation of complex congenital anatomy.^16^ From the initial experience with the use of rapid prototyping in the evaluation of the right ventricular outflow tract in candidates for percutaneous pulmonary valve implantation,^8^ novel 3D visualization have been developed to aid planning, such as VR and augmented reality.^14^

According to our results, the use of a simple 3D reconstruction such as the 3D pdf does not result in any additional benefit in identifying patients suitable for biventricular repair. This may be explained by the fact that the 3D pdf format allows a limited manipulation of the model and may result in difficult interpretation by the cardiac surgeon. Nevertheless, this format is easily portable on any device and sharable within the clinical team.

Physical 3D printed models in 1:1 scale allow a better appreciation of the spatial relationship between the interventricular communication and the great arteries, of the volume of the two outflows and of the orientation and extent of the outlet septum. However, once printed, they are not easy to modify by the user. In our study, we used rigid white nylon 3D models prepared with two cuts on the RV free wall and on the LV posterior wall to expose the intracardiac anatomy. This type of model is easier and less expensive to manufacture if compared with the flexible full models (rubber-like polyurethane filament) used in previous studies,^5^^,^^17^ yet, in this format the models were still effective in identifying the performed procedures and superior to 3D pdf and CT/CMR alone.

Virtual reality proved to be the best tool for our two evaluating surgeons to identify correctly the feasibility of biventricular repair and the need for ASO. The immersive nature of the VR experience confirmed to be an advantage for the study of complex anatomies in line with recent developments.^15^^,^^18^ The in-house VR setup was designed to provide the surgeons with the evaluation of the patient-specific model from conventional and non-conventional surgical views, to simulate the best surgical approach to the defect and to explore the intracardiac anatomy in a more comprehensive way than what is possible in the operating theatre where intracardiac anatomical assessment is limited by the surgical approach, usually from the right cardiac chambers.

Albeit a proper analysis of the costs/benefit was not part of the objectives of the study, it is interesting to draft a first comparison of the potential costs associated with each 3D modality. Provided that the costs associated with imaging and segmentation are fixed, the 3D pdf on the screen is virtually for free. In general, the file converter .stl to 3D pdf is in fact widely available in the majority of segmentation software. The cost of the materials used to print each DORV model at our Centre for this study (rigid and white) was around £100/model. This cost does not include the costs of the machine. In terms of the VR, the headset used in our setting was £300. Other associated cost would include the price for VR software. According to the EU and US regulations, this is labelled as medical device. The VR software used in this study is not commercially available yet. With the VR market growing, a proper cost/effect evaluation will be indeed necessary, but it is already sensible to expect that the costs associated with VR are lower than 3D printing.

Although small, our cohort represents a carefully selected and homogenous population of DORV with complex interventricular communications, a rather rare cardiac anomaly where the application of 3D advance visualization tools could meaningfully contribute to surgical planning. The results of this preliminary experience will be verified in future studies on larger and heterogeneous cohort of patients and conditions.

**Figure 3:**
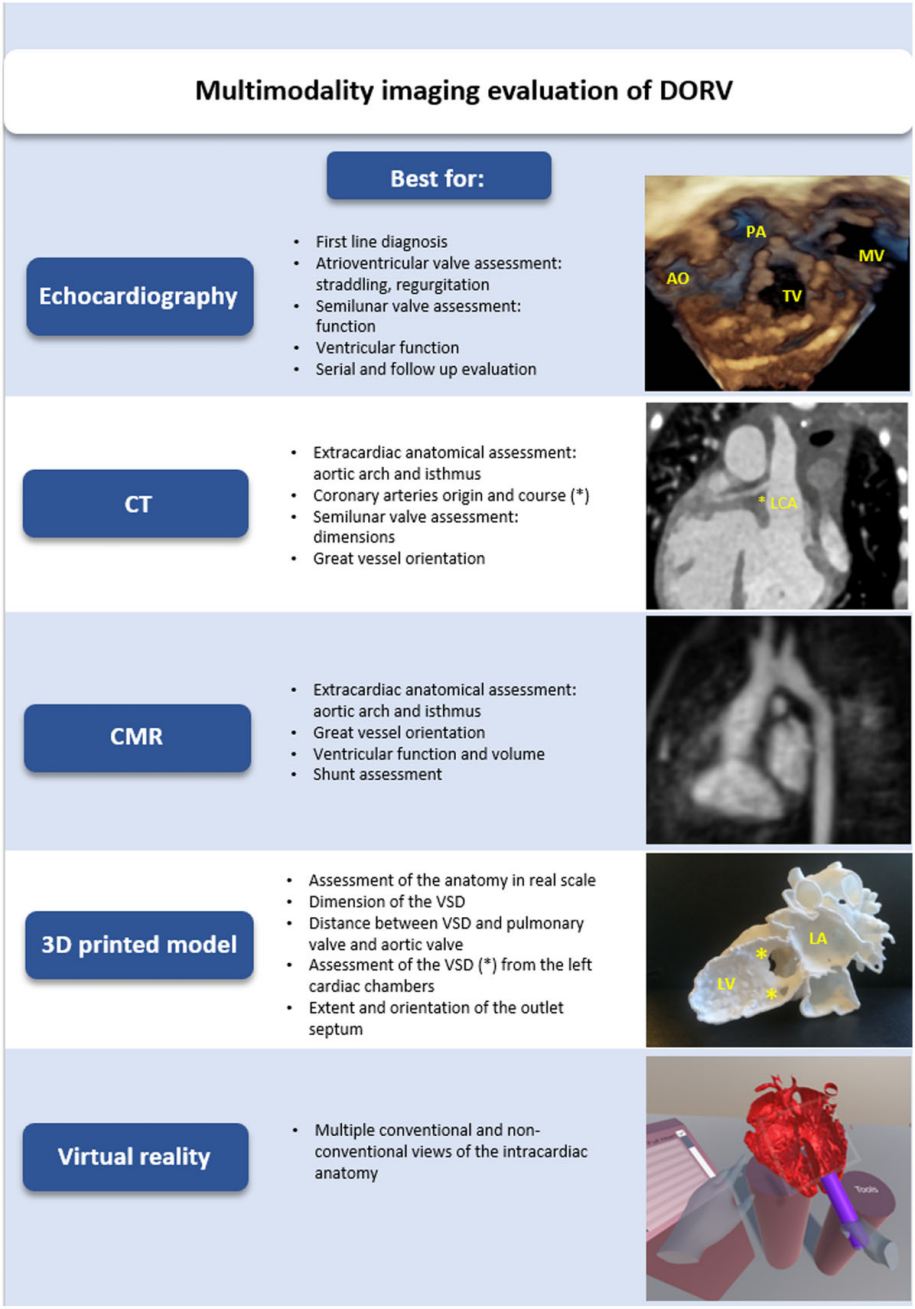
Non-invasive multimodality imaging assessment of double outlet right ventricle: three-dimensional modelling techniques like three-dimensional printing and virtual reality represent additional non-invasive imaging tools for the assessment of complex congenital cases, such as double outlet right ventricle. AO, aorta; CMR, cardiac magnetic resonance; CT, computed tomography; DORV, double outlet right ventricle; MV, mitral valve; PA, pulmoanry valve; TV, tricuspid valve; VSD, ventricular septal defect; 3D, three-dimensional.

An inherent limitation of all the 3D modelling techniques used in this study is the assessment of the atrioventricular valves. This depends on the source imaging modality (either CT or CMR), which cannot display valve structures with sufficient resolution for 3D reconstruction. Since the insertion of the tricuspid valve represents a crucial part of the surgical correction of DORV, it is recommended that 3D models fuse information from multimodality imaging information such as conventional and 3D echocardiography. In this context, 3D visualization, in particular VR, should be considered as a very valuable addition to non-invasive imaging modality (*Figure  3*).

The choices of the two evaluating surgeons were compared to actual operation performed by a senior surgeon, after joint discussion among cardiologists and surgeons and revision of clinical and imaging data. This assumption was confirmed by the fact that the original plan (i.e. biventricular repair) was not changed intraoperatively and excellent short-term results were achieved, as summarized in *Tables 2* *and 3*. Albeit the biventricular repair in patients with complex DORV and remote VSDs is the preferred option in our Institution, ultimately, it is the long-term re-intervention free survival that determines the best approach to this challenging group of patients, and this can be achieved using different surgical strategies. In addition, even a VR model will not inform about the details of surgical repair such as choice of material and patch geometry, which together with the underlying anatomy will influence freedom from re-intervention in long term.

The different advance visualization modalities were presented in consecutive order and not randomly to the surgeons, after revision of the CT or MRI data. This strategy was decided to better mimic a real-world scenario and real-world decision-making process. To date, this process starts from traditional cross-sectional imaging data and clinical information, followed by, upon request, visualization of 3D models on screen and 3D printed models. This process is likely to change with more VR opportunities. However, we decided to maintain the same order in order to isolate the potential added values of VR. A limitation of this approach, however, is the possible bias from the additional information acquired by the surgeons at each step and from each modality. In the future, we will overcome such limitation in larger studies where we will randomize the orders of presentations.

A further limitation is the fact that testing of our hypothesis was done by two senior evaluators only. We felt, however, that given the complexity of the assessed conditions, additional testing by less expert evaluators may not offer conclusive results. The findings of this pilot study will lay the basis to plan larger studies which will test our hypotheses on a wider population of respondents with different experience who will be asked to evaluate cases of various complexity.

According to our results, we can conclude that VR can potentially improve the procedural planning by offering superior information and therefore helping to select patients suitable for biventricular repair if compared to other 3D visualizations. It complements surgical strategy planning and should be ideally included in the diagnostic workup of complex DORV, alongside traditional imaging modalities (echocardiography, CT, CMR), and simulation of treatments^19^ to reach the most comprehensive evaluation possible.

Further studies are required to determine the role of VR experiences in other conditions, establish which other clinical settings may benefit from such technologies and to evaluate their additional value in training of junior surgeons.
